# The Therapeutic Value of Bone Marrow-Derived Endothelial Progenitor Cell Transplantation after Intracerebral Hemorrhage in Rats

**DOI:** 10.3389/fneur.2017.00174

**Published:** 2017-05-02

**Authors:** Rui Zhang, Jing Yang, Jingjing Yuan, Bo Song, Yan Wang, Yuming Xu

**Affiliations:** ^1^Department of Neurology, The First Affiliated Hospital of Zhengzhou University, Zhengzhou, China; ^2^Department of Urology, The First Affiliated Hospital of Zhengzhou University, Zhengzhou, China

**Keywords:** endothelial progenitor cells, intracerebral hemorrhage, blood–brain barrier, inflammation, neuroprotection, cell death

## Abstract

**Aims:**

To study the effect of endothelial progenitor cell (EPC) treatment on intracerebral hemorrhage (ICH) in rats and elucidate possible mechanisms.

**Methods:**

The rats were randomly divided into three groups: (1) EPC group: ICH + EPC, (2) phosphate-buffered saline group: ICH + PBS, and (3) sham group. EPCs were transplanted intravenously 6 h after ICH. Modified neurological severity score was used to evaluate neurological function. Blood–brain barrier (BBB) integrity was evaluated. Dead cells, inflammatory cytokines, and neuroprotective cytokines were assessed to investigate possible mechanisms.

**Results:**

The animals in the EPC group showed significant improvement in neurological function at 48 h, 72 h, and 7 days after ICH, compared with those in the PBS group. EPC transplantation significantly reduced brain edema and the number of dead cells in the hematoma boundary areas. The intensity of Evans Blue was decreased, and expression levels of zonula occluden-1 and claudin-5 were increased in the EPC group. Proinflammatory cytokines, including interferon-γ, IL-6, and TNF-α, were decreased, whereas anti-inflammatory cytokines, including transforming growth factor-β1 and IL-10, were increased in the EPC group. In addition, expression levels of brain-derived neurotrophic factor, vascular endothelial growth factor, and neurotrophic growth factor were increased following transplantation of EPCs.

**Conclusion:**

EPC transplantation could improve neurological function of ICH rats. The protective effect may be mediated by promotion of neuroprotective cytokine secretion, restoration of the BBB, reduction of cell death, and the decrease in inflammation.

## Introduction

Intracerebral hemorrhage (ICH), which manifests as brain parenchymal bleeding, accounts for approximately 10–20% of cerebral strokes (1). Patients with ICH usually have poor functional outcomes and high mortality rates. Therefore, it is considered one of the most devastating diseases (2). However, management consists primarily of symptomatic and supportive treatments. There is no effective neuroprotective treatment currently available (3). An important key pathophysiological mechanism of neurological deterioration after ICH is perihematomal edema. This is mainly caused by loss of integrity of the blood–brain barrier (BBB) and inflammatory reactions (4, 5). Therefore, reducing brain edema after ICH may be a potentially effective therapeutic method for ICH.

Endothelial progenitor cells (EPCs) are precursor cells of endothelial cells (ECs). EPCs have been shown to mobilize to the ischemic area, proliferate, and then differentiate into mature ECs. EPC therapy has advanced treatment in cardiovascular diseases and limb ischemia (6, 7). It is also a promising method for cerebral ischemic diseases (8). In an animal model of cerebral ischemia, injected EPCs could accumulate in the ischemic areas. Furthermore, they could promote recovery from neurological function deficits by attenuating cell injury through decreases in ischemia-induced apoptosis, oxidative stress, and macrophage infiltration. EPCs also participate in angiogenesis and neurogenesis, which are main factors in improvement of long-term outcomes (9). It has also been suggested that angiogenic and vasculogenic properties of EPCs might be helpful for reconstruction of a damaged BBB (10). Whether they are protective in ICH models is not known. However, clinical studies have found increased levels of circulating EPCs in ICH patients, and a higher EPC level is associated with lower blood residual volume and a better prognosis (11, 12). These results indicate that EPCs could be a new marker for hemorrhagic stroke outcomes. In addition, they could be protective following cerebral hemorrhage.

To our knowledge, only a few clinical studies have focused on the relationship between EPCs and ICH. There has been little basic research, and the mechanisms are not clear. Therefore, we used EPCs in a rat model of ICH and observed changes in neurological function. We investigated possible mechanisms including restoration of the BBB, reduction of inflammation, and a decrease in cell death.

## Materials and Methods

### Animals

We minimized their pain and the number of animals used. We purchased the adult male Sprague Dawley rats (weighing 250–280 g) from the Animal Center of Henan Province (Zhengzhou, China). The animals were housed with a 12 h light–dark cycle with free access to food and water. The rats were randomly divided into three groups: (1) EPC group: ICH + EPC, (2) phosphate-buffered saline (PBS) group: ICH + PBS, and (3) sham group.

### ICH Model

Intrastriatal injection of collagenase type VII was used to induce the rat ICH model as previously described with modifications (13). An intraperitoneal injection of 10% chloral hydrate (3 mL/kg) was used to anesthetize the rats. Then the rats were placed in a stereotaxic frame (Narishige SN-3, Tokyo, Japan) under aseptic conditions. After bregma was exposed, a 30-G needle was inserted into the striatum through a burr hole that was 0.2 mm posterior and 3.0 mm lateral to bregma, and 6.0 mm below the surface of the skull. A saline solution (3 µL) containing 0.8 U of bacterial collagenase type VII (Sigma-Aldrich, St. Louis, MO, USA) was injected over a duration of 5 min. The needle was withdrawn slowly after an additional 5 min. Bone wax was used to seal the burr hole. The scalp was then sutured. The sham-operated rats were treated simultaneously with the same method except for injection of an equivalent volume of sterile saline instead of collagenase. All rats survived the operation.

### Cell Preparation and Transplantation

Bone marrow-derived EPCs were prepared as follows (14). Briefly, the femurs and tibias of rats were isolated under sterile conditions. The ends of the long bones were cut away, and the bone marrow was collected. Density gradient centrifugation was used to isolate mononuclear cells (MNCs). The cells were then seeded onto fibronectin-coated 24-well plates. The cells were maintained in endothelial basal medium-2 (EBM-2) supplemented with EGM-2 Single-Quots (Clonetics, USA) under standard conditions (humidified atmosphere, 5% CO_2_, and 37°C). We changed the medium every 4 days, and the appearance of well-circumscribed colonies was monitored daily.

After approximately 2 weeks, the cells were characterized by expression of cell membrane antigens including endothelial antigens such as CD34, VEGFR2, and CD133. In addition, the ability of cells to take up DiI-Ac-LDL and FITC-UEA-1 was measured. The EPCs were labeled by CM-DiI (Molecular Probes, USA) before transplantation. EPCs were successively incubated for 5 min at 37°C and 15 min at 4°C in CM-DiI solution (5 µg/mL). The EPCs were then washed three times in PBS and dissolved in PBS to a final concentration of 1 × 10^7^ cells/mL. A 500-µL solution containing 5 × 10^6^ cells was injected through the femoral vein 6 h after surgery.

### Neurological Function Tests

Neurological function of the animals was evaluated by modified neurological severity score (mNSS) before and at 24 h, 48 h, 72 h, and 7 days after ICH. The mNSS is a composite score of sensory, motor, reflex, and balance tests ranging from 0 (normal) to 18 (maximal deficit) (9).

### Brain Edema

The common wet–dry method was used to evaluate brain water content at 24, 48, and 72 h after ICH as described previously with modifications. Briefly, the brain hemispheres of the rats were removed immediately after anesthetization. The ipsilateral brain hemispheres were weighed on an electronic balance immediately (wet weight) and then dried in an oven at 100°C for 24 h (dry weight). Brain water content was calculated as a percentage using the following formula: [(wet weight − dry weight)/(wet weight)] × 100% (15).

### Terminal Deoxynucleotidyl Transferase-Mediated dUTP Nick End Labeling (TUNEL) Staining

As described previously with slight modifications, dead cells including apoptotic and necrotic cells were detected at 24, 48, and 72 h after ICH by TUNEL staining (16). An *in situ* cell death detection kit (Roche, USA) was used according to the manufacturer’s instructions. The slides were analyzed with fluorescence microscopy in which the investigator was blinded to the animal groups. The number of TUNEL-positive cells was determined at a high magnification and was measured in five hemorrhage boundary zones of the ipsilateral hemisphere.

### Evans Blue (EB) Staining

Blood–brain barrier leakage was assessed by EB staining at 24, 48, and 72 h after ICH. After being anesthetized, the rats were injected with 2% EB solution (8 mL/kg, Sigma-Aldrich) through the femoral vein. Three hours later, transcardial perfusion with PBS was performed. The brain hemispheres were then removed and homogenized in *N*,*N*-dimethylformamide. The samples obtained were incubated in a 50°C water bath for 48 h and then centrifuged at 12,000 × *g* for 30 min. The supernatant was measured at 620 nm with a spectrophotometer (2,000°C, Thermo Fisher) (17).

### Western Blotting

At 72 h after ICH, the rats were killed by injection with an overdose of chloral hydrate. The brain hemispheres were harvested immediately, and total protein of the hemorrhagic hemisphere was isolated from ipsilateral brain tissues with cold RIPA buffer. A BCA protein assay kit (Dingguo, China) was used to determine protein concentration. The supernatant was subjected to SDS-polyacrylamide gel electrophoresis, and the separated proteins were transferred to a polyvinylidene difluoride filter membrane. The membranes were blocked with 5% non-fat dry milk for 1 h. Next, membranes were probed overnight with primary antibodies for zonula occluden (ZO)-1, claudin-5, brain-derived neurotrophic factor (BDNF), neurotrophic growth factor (NGF), vascular endothelial growth factor (VEGF), and insulin growth factor 1 (IGF-1) (Abcam, USA). The blots were incubated with secondary antibodies for 1 h after washing with Tris-buffered saline. An enhanced chemiluminescence kit (Solarbio, China) was used to visualize bands in immunoblots, and GAPDH (1:1,000, Santa Cruz, CA, USA) was used as the loading control.

### Enzyme-Linked Immunosorbent Assay (ELISA)

The rats were killed 72 h after ICH, brain tissues were dissected, and the following cytokine levels were quantified by ELISA: IL-10, IL-1β, IL-6, interferon (IFN)-γ, tumor necrosis factor (TNF)-α, and transforming growth factor (TGF)-β1. A microplate reader (ChemiDoc XRS+, Bio-Rad) was used for photometric measurements at 450 nm. Commercial ELISA kits (Westang, China) were used according to the manufacturer’s instructions.

### Statistical Analysis

Data are presented as mean ± SD. All data were analyzed by SPSS 13.0 software (SPSS, Chicago, IL, USA). Student’s *t* test or one-way analysis of variance was used to analyze differences between groups. A *P* < 0.05 was taken as statistically significant.

## Results

### Cell Culture and Characterization

**Figure 1:**
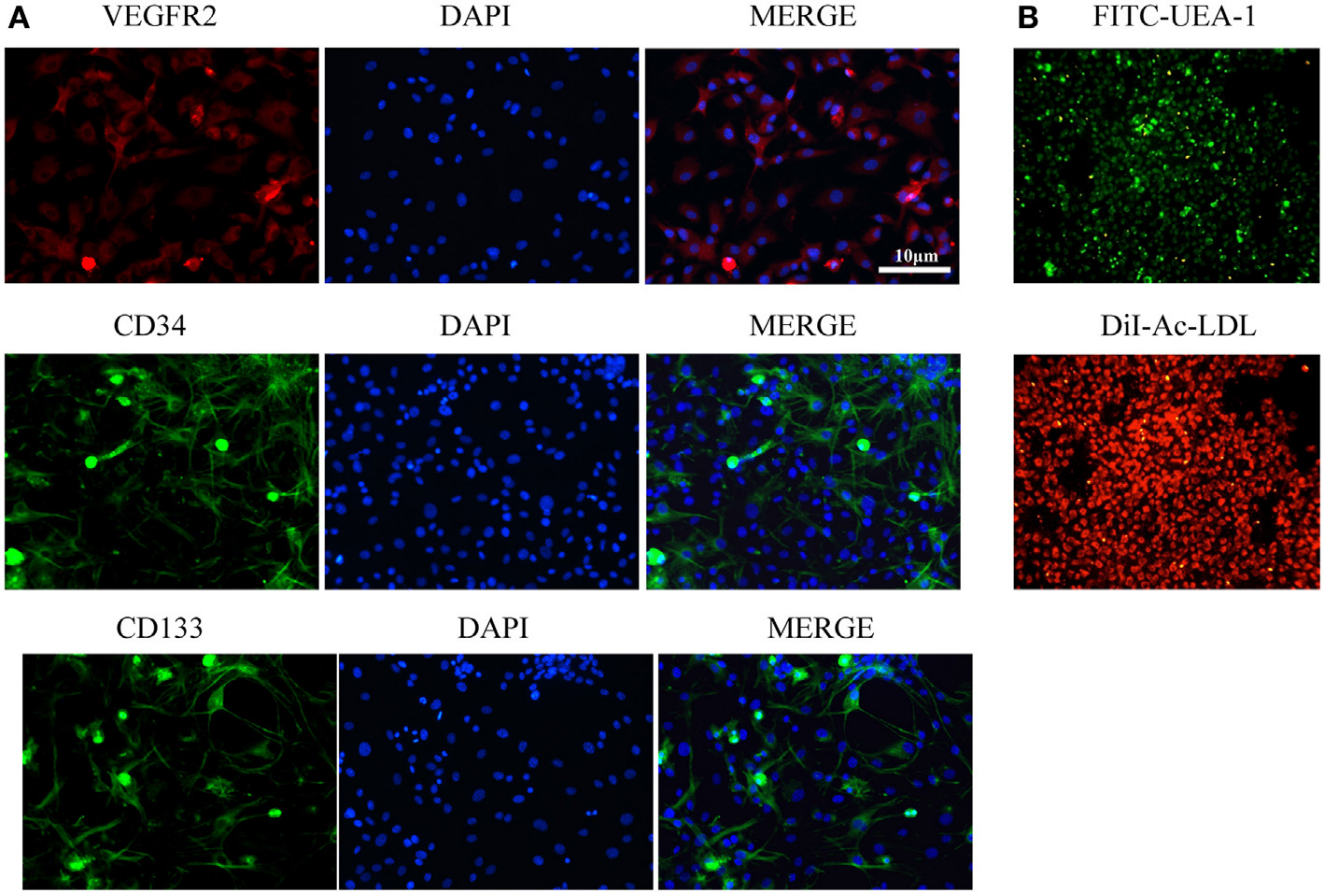
**Characterization of endothelial progenitor cells (EPCs)**. **(A)** Immunostaining showed that most attached cells expressed EPC markers VEGFR2, CD34, and CD133. **(B)** Double staining with FITC-UEA-1 and DiI-Ac-LDL showed that 95% of EPCs could uptake Ac-LDL and bind endothelial cell-specific lectin UEA. Scale bar = 10 μm.

Endothelial basal medium-2 was used to culture MNCs from rat bone marrow for 14 days to obtain EPCs as previously described. The putative EPCs attached to the plates and then assumed a spindle-like shape. Double staining with DiI-Ac-LDL and FITC-UEA-1 indicated that 95% of the EPCs could uptake Ac-LDL and bind EC-specific lectin UEA. Immunostaining showed that most attached cells expressed EPC markers CD34, VEGFR2, and CD133 (Figure 1).

### Behavioral Tests

**Figure 2:**
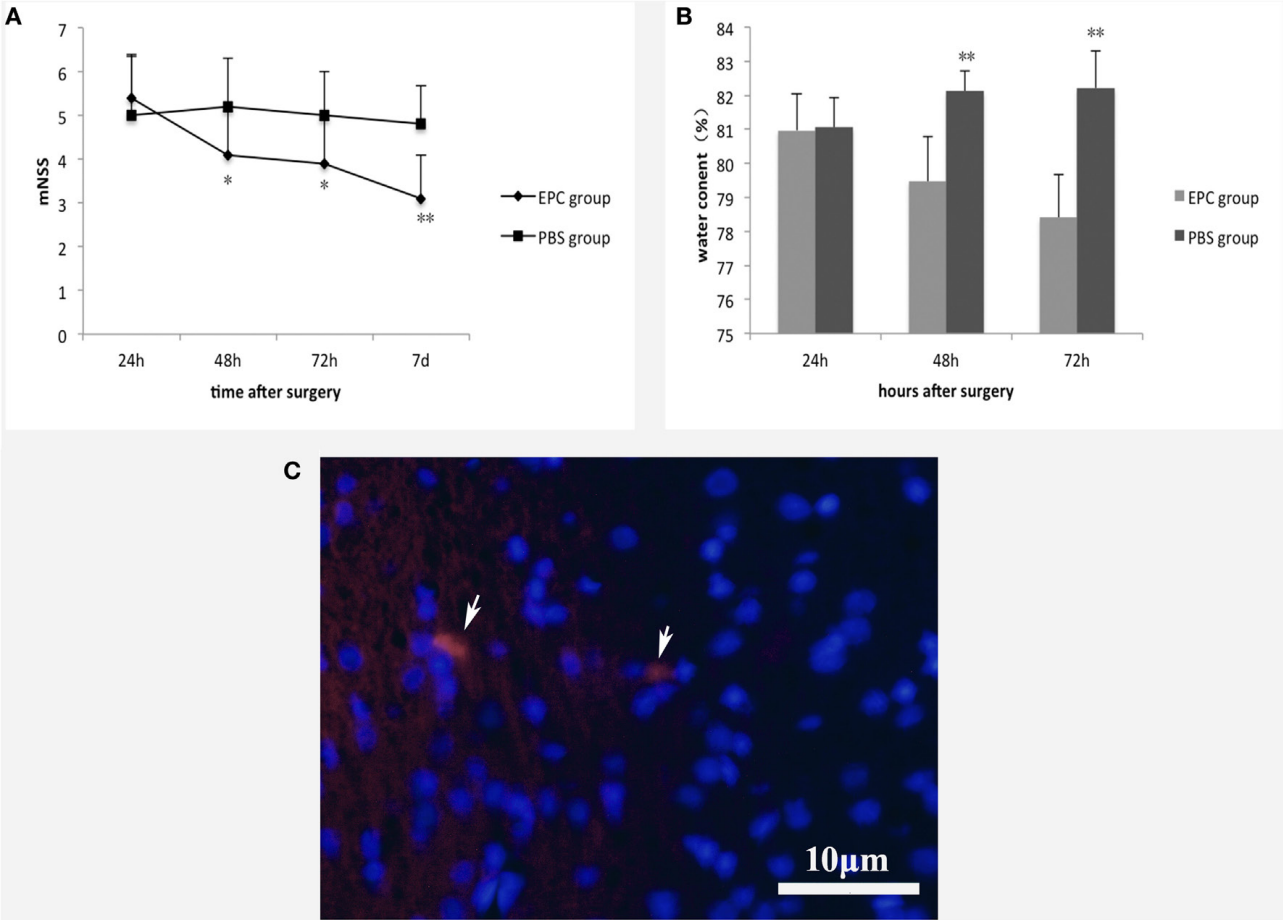
**Effect of EPC transplantation on neurological function and brain edema**. **(A)** Neurological function was evaluated by mNSS 24 h, 48 h, 72 h, and 7 days after ICH. Rats in the EPC group showed significant improvement in neurological function at 48 h, 72 h, and 7 days after ICH compared with those in the PBS group. **(B)** Brain water content was evaluated 24, 48, and 72 h after ICH. EPC transplantation significantly reduced brain water content compared with the PBS group at 48 and 72 h after ICH. **(C)** Distribution of transplanted EPCs. At 72 h after transplantation, CM-DiI-labeled cells were detected by fluorescence microscopy. Few labeled cells were detected in the peripheral area of the hematoma. Scale bar = 10 μm. For the mNSS test, *n* = 10 at each time point per group. For the brain water content test, *n* = 6 at each time point per group (*P < 0.05, **P < 0.001). mNSS, modified neurological severity score; EPCs, endothelial progenitor cells; PBS, phosphate-buffered saline; ICH, intracerebral hemorrhage.

Neurological function of the animals was compared between the EPC and PBS groups before cell transplantation, and there was no significant difference in mNSS between groups. In contrast, at 48 h (*P* < 0.05), 72 h (*P* < 0.05), and 7 days (*P* < 0.001) after ICH, animals in the EPC group showed significant improvement in neurological function compared with those in the PBS group (Figure 2A). The distribution of transplanted EPCs was assessed 72 h after transplantation using fluorescence microscopy to detect CM-DiI-labeled cells. Few labeled cells were detected in the peripheral area of the hematoma (Figure 2C), and no labeled cells were detected in the vessel walls. No labeled EPCs were observed in the PBS-treated group.

### Water Content

We measured brain water content to assess brain edema in hemorrhagic hemispheres. Animals in the PBS group had much higher brain water content compared with those in the sham group. After ICH, EPC transplantation significantly reduced brain water content compared with the PBS group at 48 h (*P* < 0.001) and 72 h (*P* < 0.001; Figure 2B).

### TUNEL Staining

**Figure 3:**
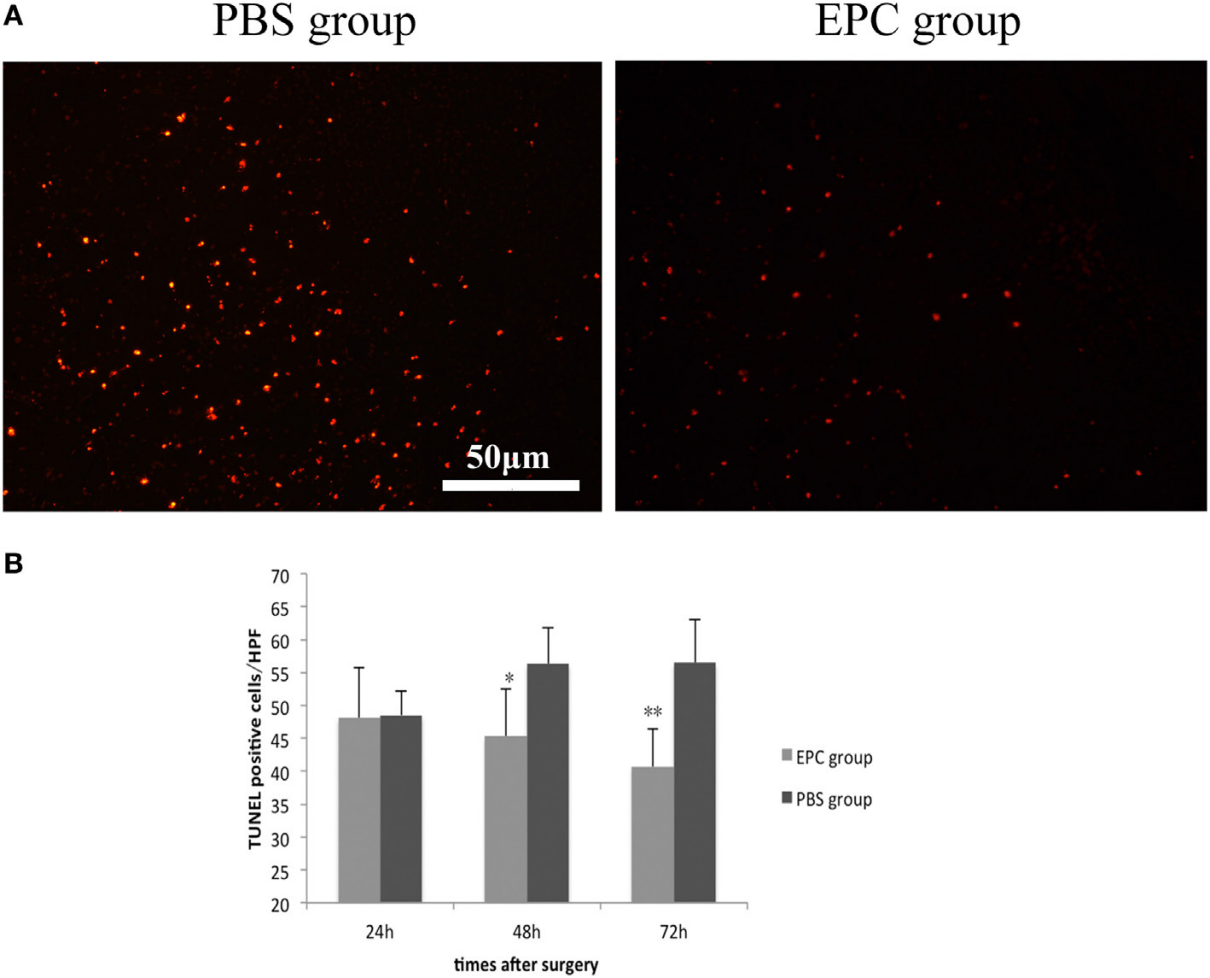
**Effect of endothelial progenitor cell (EPC) transplantation on cell death**. TUNEL staining was conducted to investigate dead cells 24, 48, and 72 h after intracerebral hemorrhage (ICH) in the peripheral area of the hematoma. The number of TUNEL-positive cells in the boundary area was decreased at 48 and 72 h after ICH in the EPC group compared with the phosphate-buffered saline group, *n* = 6 at each time point per group (*P < 0.05, **P < 0.001). TUNEL, terminal deoxynucleotidyl transferase-mediated dUTP nick end labeling. Scale bar = 50 μm **(A,B)**.

Terminal deoxynucleotidyl transferase-mediated dUTP nick end labeling staining was conducted to investigate dead cells including apoptotic and necrotic cells at 24, 48, and 72 h after ICH in the peripheral area of the hematoma in both EPC and PBS groups. The number of TUNEL-positive cells in the boundary area was decreased at 48 h (*P* < 0.05) and 72 h (*P* < 0.001) after ICH in the EPC group compared with the PBS group (Figure 3).

### BBB Integrity

**Figure 4:**
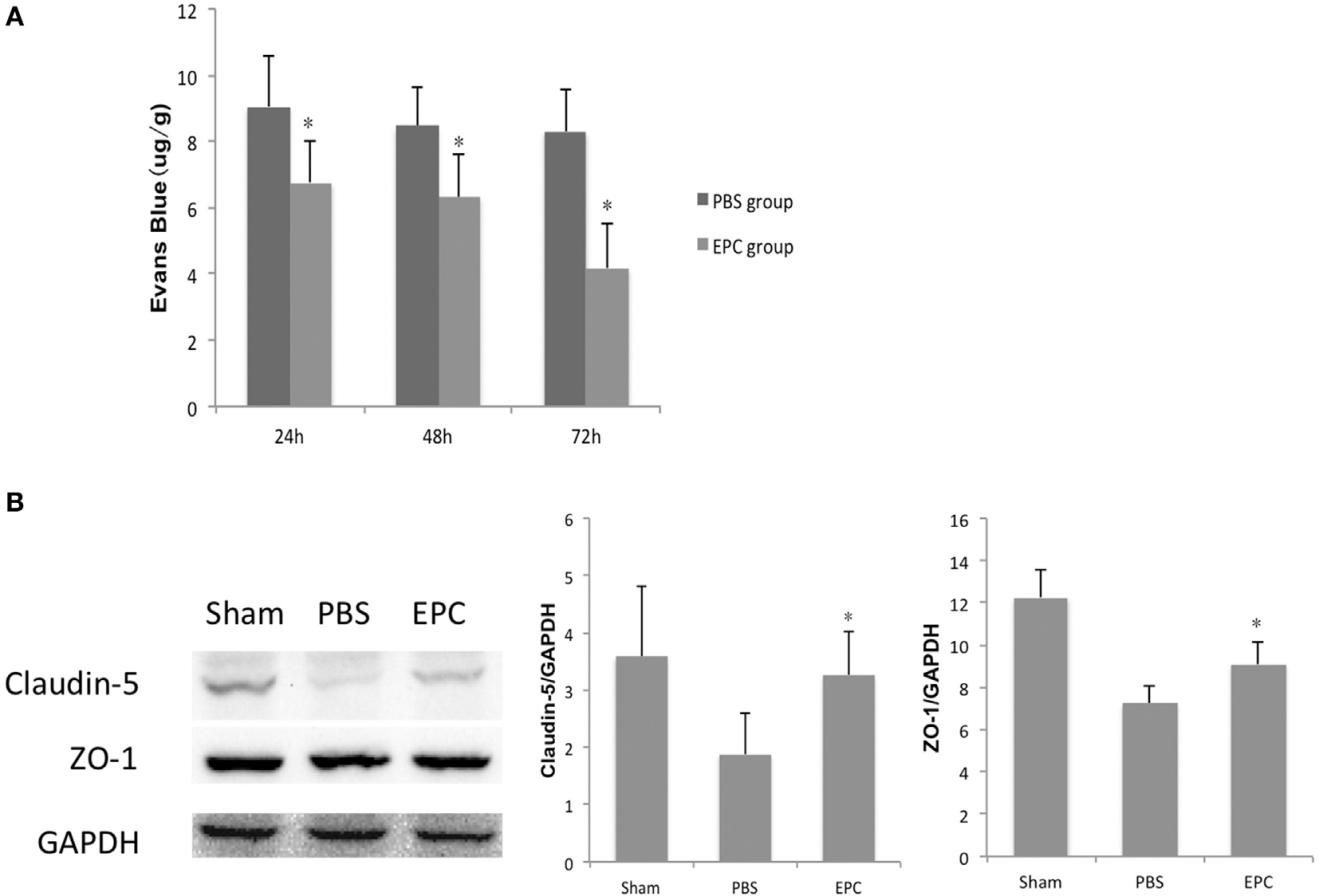
**Effect of endothelial progenitor cell (EPC) transplantation on restoration of the BBB**. **(A)** BBB leakage was assessed by Evans Blue (EB) staining 24, 48, and 72 h after intracerebral hemorrhage (ICH). EB intensity decreased at all three time points in the EPC group. **(B)** Expression of tight junction proteins, including claudin-5 and zonula occluden (ZO)-1, was studied by Western blot analysis 72 h after ICH. Compared with the phosphate-buffered saline group, expression levels of claudin-5 and ZO-1 increased in the EPC group, *n* = 6 at each time point per group (*P < 0.05). BBB, blood–brain barrier.

Evans Blue extravasation was measured to assess integrity of the BBB at 24, 48, and 72 h after ICH. The intensity of EB determined by spectrofluorometry was decreased in the EPC group compared with the PBS group at all three time points (*P* < 0.05), which reflected reduced BBB leakage in the EPC group (Figure 4A). Expression levels of tight junction proteins, including ZO-1 and claudin-5, were studied by Western blot analysis to evaluate microvascular integrity at 72 h after ICH. Expression levels of ZO-1 (*P* < 0.05) and claudin-5 (*P* < 0.05) were increased in the EPC group compared with the PBS group (Figure 4B).

### Cytokine Profiling

**Figure 5:**
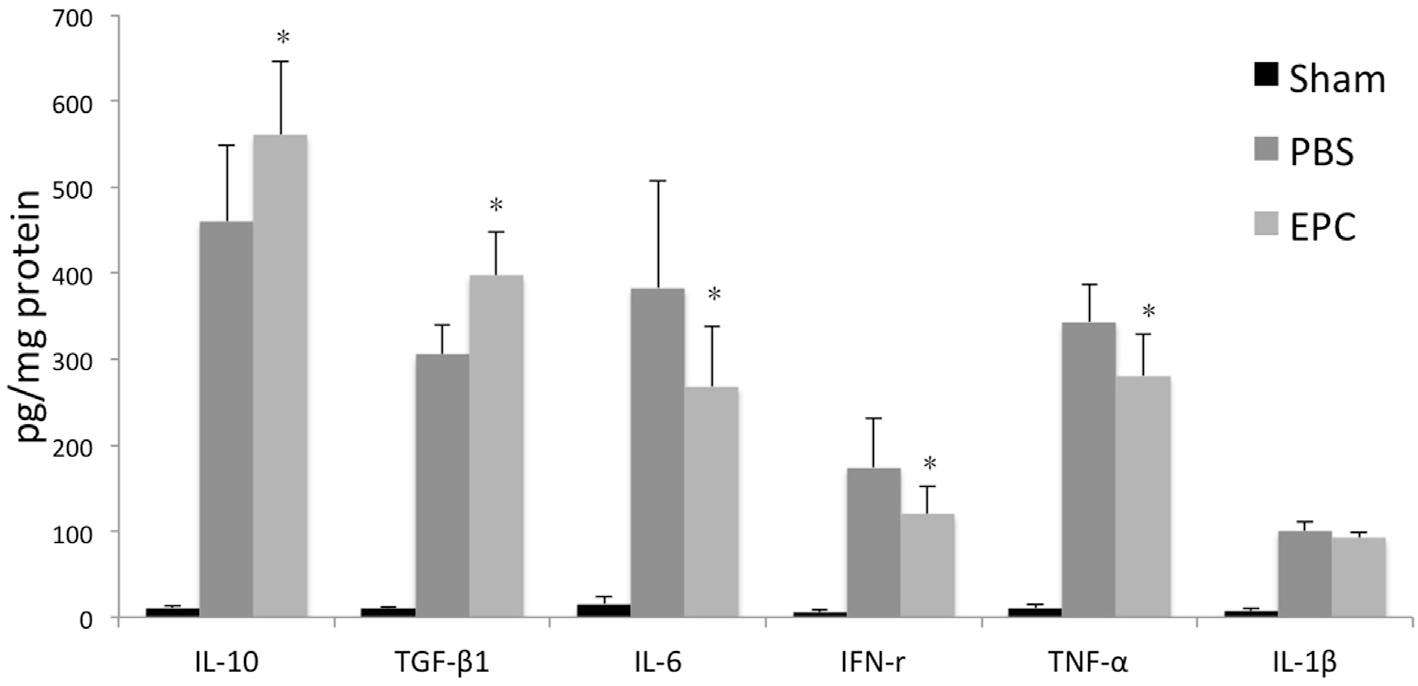
**Influence of endothelial progenitor cell (EPC) transplantation on inflammatory-related cytokines**. Expression levels of inflammatory-related cytokines in the hemorrhagic hemisphere were measured by enzyme-linked immunosorbent assay 72 h after intracerebral hemorrhage. Levels of pro-inflammatory cytokines, including IL-6, interferon-γ, and TNF-α, decreased after EPC transplantation. In contrast, levels of anti-inflammatory cytokines, including IL-10 and transforming growth factor-β1, increased in the EPC group. *n* = 6 at each time point per group (*P < 0.05).

**Figure 6:**
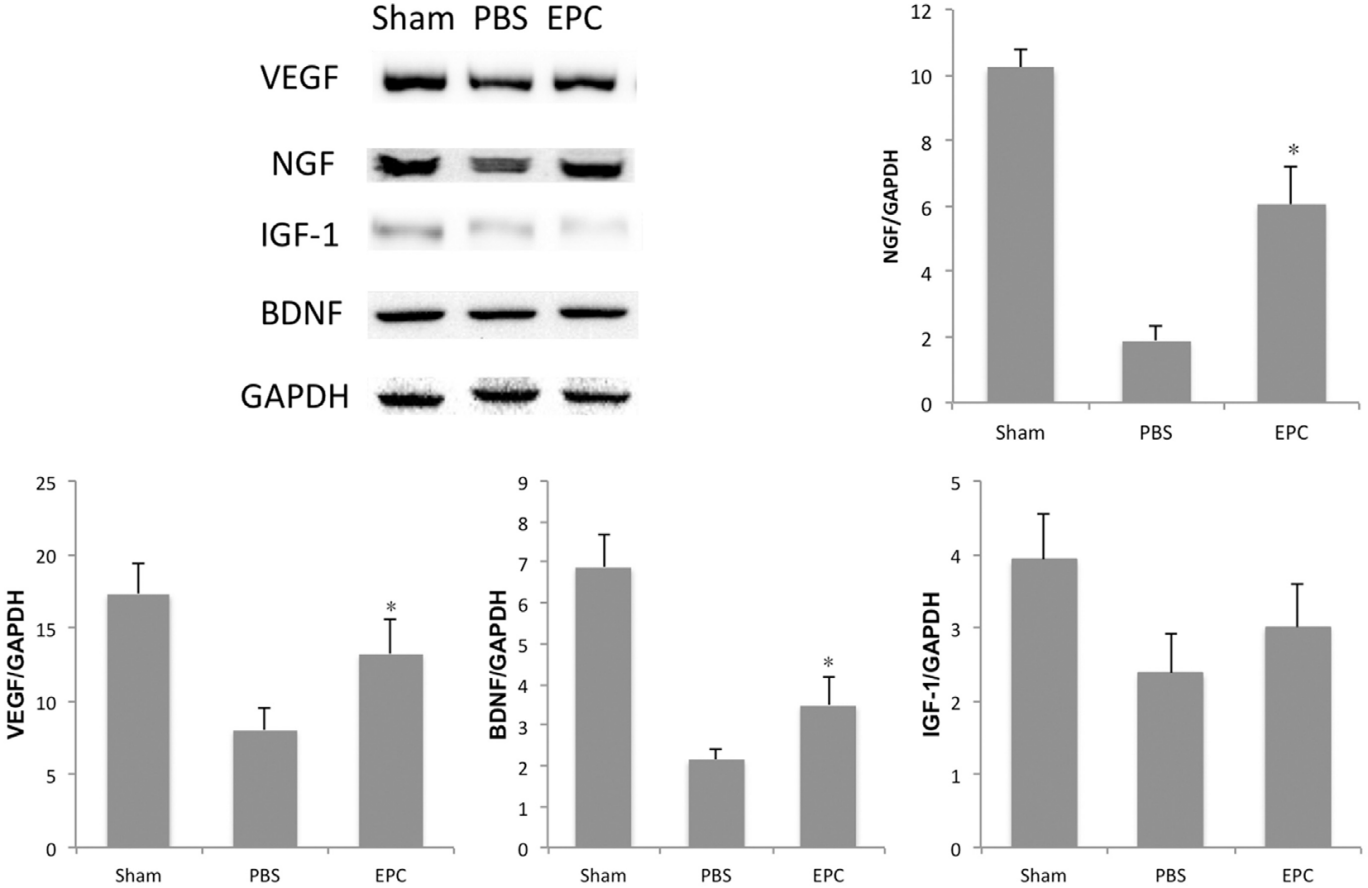
**Influence of endothelial progenitor cell (EPC) transplantation on protective cytokines**. At 72 h after intracerebral hemorrhage, expression levels of protective cytokines, including VEGF, BDNF, NGF, and IGF-1, were measured in brain homogenates by Western blot analysis. Expression levels of VEGF, BDNF, and NGF increased in the EPC group compared with the phosphate-buffered saline group. *n* = 6 at each time point per group (*P < 0.05). BDNF, brain-derived neurotrophic factor; VEGF, vascular endothelial growth factor; NGF, neurotrophic growth factor; IGF-1, insulin growth factor 1; GAPDH, glyceraldehyde 3-phosphate dehydrogenase.

Expression of inflammatory-related cytokines in the hemorrhagic hemisphere was examined by ELISA 72 h after ICH to investigate the effects of EPCs on inflammation. Levels of pro-inflammatory cytokines, including IL-6, TNF-α, and IFN-γ, were decreased after EPC transplantation (*P* < 0.05). However, expression levels of IL-1β did not change significantly. Levels of anti-inflammatory cytokines, including TGF-β1 and IL-10, were increased in the EPC group (*P* < 0.05; Figure 5). Expression of protective cytokines, including VEGF, BDNF, NGF, and IGF-1, in homogenates from the ipsilateral hemisphere was evaluated by Western blot analysis. Transplantation of EPCs increased levels of NGF, VEGF, and BDNF at 72 h after ICH compared with the PBS group (*P* < 0.05). Expression of IGF-1 did not increase significantly (Figure 6).

## Discussion

Intracerebral hemorrhage is one of the most devastating diseases. To date, there is no effective neuroprotective treatment (18). Stem cell therapy has drawn much attention. Researchers have shown that mesenchymal stem cell (MSC) transplantation can decrease BBB leakage, enhance nerve regeneration of the cortical spinal tract, and improve recovery of neurological function in ICH rats (15, 19). As the precursor cells of endothelial cells, EPCs have been widely used to study therapeutic effects and possible mechanisms in ischemic stroke models. It has been shown that EPCs can elevate the prognosis of ischemic stroke by repairing damaged ECs, promoting repair of injured nerves, and enhancing formation of new blood vessels (20, 21). Because of vasculogenic and angiogenic properties, EPCs can contribute to reconstitution of the disrupted BBB, which is an important pathophysiological factor of cerebral vascular injury (22, 23). In this study, we investigated effects of EPCs on neurological function in ICH rats and possible underlying mechanisms.

The EPCs used in our research were isolated from rat bone marrow and characterized by specific markers CD34, VEGFR2, and CD133, as previously described (21, 24). The cells were also characterized by their ability to uptake Ac-LDL and UEA. After being labeled by CM-DiI, EPCs were injected intravenously. Results indicated that EPC transplantation could attenuate neurological deficits of the animals. Furthermore, hemorrhage-induced brain edema was decreased, which may have played a role in improved neurological function of the animals.

Dysfunction of the BBB is one of the most important pathophysiological factors for edema formation after ICH (22). Therefore, changes in BBB integrity after cell transplantation were examined using EB staining to measure water permeability. Results showed that BBB leakage increased after ICH. However, after EPC transplantation, EB staining intensity decreased, which indicated restoration of the BBB. Tight junction proteins are composed of claudins, occludin, ZO, and other molecules, and their assembly determines extracellular permeability of water soluble molecules across the BBB (25–27). They are key elements in BBB integrity. Researchers have shown that induced expression of tight junction proteins could accelerate reestablishment of the BBB (23, 25). Chen et al. demonstrated that transplantation of MSCs could increase levels of ZO-1 and claudin-5, as well as attenuate brain edema and BBB leakage in a rat ICH model (15). In the present study, we measured expression levels of claudin-5 and ZO-1 and found that expression of these tight junction proteins decreased after cerebral hemorrhage, which indicated BBB disruption. Expression of tight junction proteins in brain tissues increased significantly after EPC transplantation compared with the PBS group. This was consistent with decreased brain edema and improved neurological function. Inflammatory reaction is one of the most important pathological mechanisms in ICH (28), and BBB restoration is closely related to neuronal inflammation (29, 30). We found that EPCs could decrease pro-inflammatory cytokines, such as IFN-γ, IL-6, and TNF-α, and increase anti-inflammatory cytokines such as TGF-β1 and IL-10.

Because neuronal apoptosis and necrosis is a predominant process of cerebral injury, many studies have focused on apoptotic mechanisms in delayed tissue loss. Wang et al. found that MSCs could improve neural function and reduce hemorrhage volume by elevating expression of antiapoptotic proteins, including BDNF and G-CSF, in a rat ICH model (31). Deng et al. showed that bone marrow-derived mesenchymal stem cells could reduce neuronal apoptosis by releasing VEGF in a rat ischemic stroke model (32). The antiapoptotic effect of EPCs has been discussed in ischemic stroke models in several reports. Qiu et al. found that EPCs could decrease caspse-3 activity, upregulate BCL-2 expression, and thus protect neurons from cerebral ischemia and reperfusion injury in rats (33). Moubarik et al. suggested that inhibition of apoptosis by EPCs was an effective intervention to attenuate neurological injury after middle cerebral artery occlusion. The underlying mechanisms may include upregulation of neural protective cytokines, such as IGF-1, and downregulation of proBDNF, which is a pro-inflammatory factor (9). In our research, hemorrhage-induced cell death including apoptosis and necrosis was assessed by TUNEL. The number of TUNEL-positive cells in the hematoma boundary was significantly increased after cerebral hemorrhage, and it was decreased after EPC transplantation compared with the PBS-treated group at 48 and 72 h after hemorrhage. Cytokines, including VEGF, BDNF, IGF-1, and NGF, were examined, and results showed that expression levels of VEGF, BDNF, and NGF increased after EPC transplantation. However, expression of IGF-1 did not increase significantly.

Endothelial progenitor cells can mobilize to the ischemic area and directly participate in neovascularization (34, 35). Thus, mobilization of EPCs to the area surrounding the hematoma was also investigated. Before transplantation, the cells were labeled by CM-DiI, a carbocyanine membrane probe. At 72 h after surgery, the brain tissue of the rats was dissected and observed under a fluorescence microscope. Red fluorescence could be detected in the area surrounding the hematoma in the EPC group. However, consistent with previous reports, we did not observe labeled cells in the vessel walls, which might indicate that direct neovascularization is not the main mechanism underlying the protective effects of EPCs. As discussed in other studies, EPCs have two subtypes: early EPCs and late EPCs. In the present study, the EPCs were cultured for 14 days, and CD133 staining was positive. This suggests that the cells used in the present study were early EPCs, which contributed to the neuroprotection by secreting protective cytokines (36).

## Conclusion

Endothelial progenitor cell transplantation can improve neurological function of ICH rats. The protective effects may be related to promotion of neuroprotective cytokine secretion, restoration of the BBB, reduction of cell death, and the decrease in inflammation. Although determining the underlying mechanisms requires further investigation, our results suggest that transplantation of EPCs is a promising method for treatment of ICH patients.

## Ethics Statement

This study was carried out in accordance with the recommendations of the Zhengzhou University Ethics Committee. The protocol was approved by the Zhengzhou University Ethics Committee.

## Author Contributions

RZ and JY contributed to the design of the work, cell transplantation and characterization, cell labeling, interpretation of the data, and manuscript writing. JjY contributed to the animal model establishment, TUNEL staining, ELISA, and manuscript revising. BS contributed to the cell transplantation, neurological functional evaluation, and manuscript revising. YW contributed to the cell transplantation, Western blotting, and manuscript revising. YX contributed to the conception and design of the work, analysis and interpretation of the data, and manuscript revising.

## Conflict of Interest Statement

The authors declare that the research was conducted in the absence of any commercial or financial relationships that could be construed as a potential conflict of interest.
